# Editorial: COVID and autism 2023: lessons learnt and future directions for research

**DOI:** 10.3389/fpsyt.2024.1476002

**Published:** 2024-08-28

**Authors:** Georgina Perez Liz, Michaela DuBay, Cecilia Montiel-Nava

**Affiliations:** ^1^ A.J. Drexel Autism Institute, Drexel University, Philadelphia, PA, United States; ^2^ School of Education and Human Development, University of Virginia, Charlottesville, VA, United States; ^3^ Department of Psychological Science, College of Liberal Arts, The University of Texas Rio Grande Valley, Edinburg, TX, United States

**Keywords:** autism, COVID-19, caretakers, service provision, preparedness

Over four years ago, the coronavirus pandemic sent many countries into national lockdowns, severely restricting social interaction and professional, academic, health, and leisure activities alike. The virus itself infected hundreds of millions of people, yet the wide ranging indirect impacts were felt almost universally. Those with existing conditions were impacted in unique ways. Globally, autistic people and their caregivers reported significant reduction in mental health (1–3), and reductions in service access which led to increases in challenging behaviors (4) during the acute stages of the pandemic. Impacts of the later stages of the pandemic, including phases of masking requirements, continued restrictions to non-essential social interactions, new waves of variants, and continued risk to physical health are still being explored (5).

Fully understanding both the acute and long-term impacts of the COVID-19 pandemic on the lives of autistic individuals and their caregivers will help to inform preparedness and response to future public health emergencies. This Research Topic aims to highlight evidence on how the COVID-19 pandemic has affected and continues to affect autistic individuals globally as well as to provide insights into research and interventions on how this can be tackled going forwards.

We anticipate that research presented will be able to translate to best practice applications in clinical and public health settings. Within this Research Topic, we present a range of articles including seven original research articles, one brief research report, and one opinion article. This Research Topic provides new evidence from across countries and continents on what we have learned about COVID-19 and autism since the beginning of the pandemic, including the physical and mental impacts of COVID-19 on the autistic population, the effects of lockdowns and social distancing on autistic individuals, and the impact that these aspects had and continue to have on care provided for autistic individuals. Perspectives are presented from Argentina, Brazil, Chile, the Dominican Republic, Italy, Mexico, Poland, Portugal, Qatar, Saudi Arabia, Uruguay, Venezuela, and the United States, which allows for a broad representation of global impacts.

The articles presented in this Research Topic highlighted important challenges faced by the autistic population, along with areas of opportunity to improve care during global health emergency situations. Several key themes emerged. Firstly, service delivery disruptions and access challenges were identified across essential services and supports for the autistic community. These included special education, speech and occupational therapies, applied behavior analysis, and mental health services (Gatica-Bahamonde et al.; Pokoski et al.; Tsai and Bhat). School closures and transition to remote learning were particularly challenging, and recovery from these situations continues to be slow and unequal across different demographic groups (Tsai and Bhat).

Secondly, caregivers of autistic children and adults faced increased stress and burden (Sousa et al.; Pokoski et al.), sometimes resulting in measurably poor mental health outcomes. In researching challenges and difficulties, we aimed to present studies that also discussed areas of strength, such as the study by Sousa et al., which found that family cohesion was a key protective factor against caregiver depression.

Thirdly, reports of behavioral and emotional challenges in the autistic population were a ubiquitous finding (Gatica-Bahamonde et al.; Pokoski et al.). Changes to routines and reduced access to services likely exacerbated these issues. Eating behaviors and routines were also areas negatively impacted by the COVID-19 pandemic, as reported by caregivers (Alharbi).

Fourthly, we have also learned that there were important disparities in the impact of the COVID-19 pandemic, and the importance of evaluating specific variables according to the research context. Pokoski et al. found that families of lower socioeconomic status, racial/ethnic minorities, and those in rural areas experienced greater service disruptions and financial distress. Tsai and Bhat also noted disparities in service access and recovery based on age, income, race/ethnicity and geographic location. In contrast, Alshaban et al. included hired help in the home as an analysis variable of the measured outcomes, since it is relevant in the cultural context of Qatar.

Finally, the rapid shift to telehealth and remote service delivery, from screening to intervention, presented both challenges and opportunities. While some services like screening adapted well to virtual formats (Gatica-Bahamonde et al.), others, such as occupational therapy, were more difficult to deliver remotely (Tsai and Bhat).

Although the World Health Organization (WHO) declared the end of the Public Health Emergency of International Concern (PHEIC) status for COVID-19 on May 5, 2023 (6) knowledge related to its impact in the life of people around the globe is still being uncovered. COVID-19 is no longer considered a global health emergency, though it remains a concern and requires ongoing management and vigilance. Lockdown measures, transition to online educational and health related services or their suspension, among others, were particularly challenging for autistic individuals and their families (7).

The articles in this Research Topic have cultivated reimagination of the challenges and possible solutions to the needs of autistic people, and highlight the significant impact that sudden changes in routine and access to services can have on the mental health of autistic individuals. As research shows disruptions in daily routines and reduced access to essential services during the pandemic exacerbated stress, anxiety, and other mental health issues among autistic individuals (Bieczek et al.; Montenegro et al.).

Moving forward, professionals and families can leverage these insights to better prepare for unforeseen situations. Implementing strategies such as maintaining as much routine as possible, utilizing virtual support services, and creating structured plans for potential disruptions can mitigate adverse mental health effects (Failla et al.; 8). Additionally, developing clear communication channels and providing social stories or visual schedules can help autistic individuals understand and adapt to changes, reducing anxiety and promoting stability (9). By applying these lessons learned during the pandemic, professionals and families can enhance their resilience and readiness for future interruptions or health crises.

Having learned from the experiences of adjusting services and measuring mental health issues in the autistic community across the globe, future research should focus on developing and evaluating interventions specifically designed to support autistic individuals and their families during times of disruption and crisis. For instance, more studies could explore the effectiveness of telehealth services in providing care starting at early detection, and continuous support and therapy, examining which modalities are most beneficial (10). In this way, when autistic individuals need to transition to this modality of service, they could have robust and evidence-based alternatives. Additionally, longitudinal research is needed to understand the long-term mental health impacts of pandemic-related disruptions on autistic individuals, particularly in terms of resilience and coping mechanisms (11). Investigating the role of personalized, adaptive routines and the use of technology to create virtual support networks may also provide valuable insights (12).

Furthermore, it is crucial to include the perspectives of autistic individuals and their families in research to ensure that interventions are tailored to their specific needs and preferences (13). By addressing these areas, future research can contribute to the development of more effective strategies to support the mental health and well-being of autistic individuals of all ages, abilities, and nationalities, during unforeseen circumstances.
